# Fusion Genes in Prostate Cancer: A Comparison in Men of African and European Descent

**DOI:** 10.3390/biology11050625

**Published:** 2022-04-20

**Authors:** Rebecca Morgan, Dulcie Keeley, E. Starr Hazard, Emma H. Allott, Bethany Wolf, Stephen J. Savage, Chanita Hughes Halbert, Sebastiano Gattoni-Celli, Gary Hardiman

**Affiliations:** 1 Faculty of Medicine, Health and Life Sciences, School of Biological Sciences, Institute for Global Food Security (IGFS), Queen’s University Belfast, (QUB), Belfast BT9 5DL, UK; rmorgan21@qub.ac.uk (R.M.); dkeeley02@qub.ac.uk (D.K.); 2 Academic Affairs Faculty, Medical University of South Carolina (MUSC), Charleston, SC 29425, USA; hazardes3@gmail.com; 3 Patrick G. Johnston Centre for Cancer Research, QUB, Belfast BT9 7AE, UK; e.allott@qub.ac.uk; 4 Department of Public Health Sciences, Medical University of South Carolina (MUSC), Charleston, SC 29425, USA; wolfb@musc.edu; 5 Department of Urology, Medical University of South Carolina (MUSC), Charleston, SC 29425, USA; savages@musc.edu; 6 Ralph H. Johnson VA Medical Center, Charleston, SC 29425, USA; sebastiano.gattoni-celli@bms.com; 7 Department of Population and Public Health Sciences, University of Southern California, Los Angeles, CA 90033, USA; hughesha@musc.edu; 8 Norris Comprehensive Cancer Center, University of Southern California, Los Angeles, CA 90033, USA; 9 Department of Radiation Oncology, Medical University of South Carolina (MUSC), Charleston, SC 29425, USA; 10 Department of Medicine, Medical University of South Carolina (MUSC), Charleston, SC 29425, USA

**Keywords:** prostate cancer, fusion genes, African American, African descent, European American, RNA-sequencing, biomarkers

## Abstract

**Simple Summary:**

Men of African origin have a 2–3 times greater chance of developing prostate cancer than those of European origin, and of patients that are diagnosed with the disease, men of African descent are 2 times more likely to die compared to white men. Men of African origin are still greatly underrepresented in genetic studies and clinical trials. This, unfortunately, means that new discoveries in cancer treatment are missing key information on the group with a greater chance of mortality. A fusion gene is a hybrid gene formed from two previously independent genes. Fusion genes have been found to be common in all main types of human cancer. The objective of this study was to increase our knowledge of fusion genes in prostate cancer using computational approaches and to compare fusion genes between men of African and European origin. This identified novel gene fusions unique to men of African origin and suggested that this group has a greater number of fusion genes.

**Abstract:**

Prostate cancer is one of the most prevalent cancers worldwide, particularly affecting men living a western lifestyle and of African descent, suggesting risk factors that are genetic, environmental, and socioeconomic in nature. In the USA, African American (AA) men are disproportionately affected, on average suffering from a higher grade of the disease and at a younger age compared to men of European descent (EA). Fusion genes are chimeric products formed by the merging of two separate genes occurring as a result of chromosomal structural changes, for example, inversion or trans/cis-splicing of neighboring genes. They are known drivers of cancer and have been identified in 20% of cancers. Improvements in genomics technologies such as RNA-sequencing coupled with better algorithms for prediction of fusion genes has added to our knowledge of specific gene fusions in cancers. At present AA are underrepresented in genomic studies of prostate cancer. The primary goal of this study was to examine molecular differences in predicted fusion genes in a cohort of AA and EA men in the context of prostate cancer using computational approaches. RNA was purified from prostate tissue specimens obtained at surgery from subjects enrolled in the study. Fusion gene predictions were performed using four different fusion gene detection programs. This identified novel putative gene fusions unique to AA and suggested that the fusion gene burden was higher in AA compared to EA men.

## 1. Introduction

Among men, prostate cancer (PC) is the most diagnosed cancer and a major cause of mortality, especially in those living western lifestyles [1,2]. Incidence and mortality rates of PC increase with age, with a higher rate observed in African American (AA) men compared to those of European descent (EA) [3]. PC is a heterogeneous disease that has a slow progression compared to other cancers, typically remaining latent for up to 20 years [4]. Increases in PC incidence have been reported since 1990 in both developed and developing countries [5]. This is due to the increased use of prostate-specific antigen (PSA) screening, which measures PSA levels in the blood [6]. Serum PSA screening is a general approach used to diagnose patients with PC. It can successfully detect PC in its early stages, allowing for earlier therapeutic intervention to reduce mortality rates [7]. It remains controversial whether widespread screening is beneficial, as this approach has resulted in the excessive diagnosis of PC cases which may otherwise have remained clinically undetected and insignificant [7,8]. 

Knowledge of PC genomics has grown in recent years, allowing several race-specific variations to be identified, including polymorphisms, gene mutations, and epigenetic changes [9]. Genetic abnormalities are common in cancers, the most prevalent being the chromosomal rearrangements that lead to the formation of fusion genes, for example, through deletions, inversions, and amplifications [3,10]. This results in a chimeric gene product being formed from the two separate genes involved [11]. The first of these to be discovered was the fusion of the break point cluster (*BRC*) and Abelson murine leukemia viral oncogene homolog 1 (*ABL1*) genes, better known as the Philadelphia chromosome [12]. It has been detected in 95% of chronic myelogenous leukemia patients, and since that initial discovery many fusion genes have been identified as drivers of cancer through deregulation, loss of function, or the formation of oncogenic proteins [13,14]. The discovery of gene fusions in cancer has increased with the advent of high throughput sequencing. 

The prostate-specific androgen response gene *TMPRSS2* and its fusion with the ETS-related gene (*ERG*) have been extensively studied in PC [15,16]). It is the leading driver of *ERG* overexpression in PC, which is associated with the activation of biological pathways and their constituent proteins that promote cancer progression [16]. Frequencies of genetic alterations that are potential drivers of cancer vary between populations of different ancestries. For example, although the total burden of transcript fusion events was not found to differ by genetic ancestry, the *TMPRSS2-ERG* fusion was more frequently observed in EAs than AAs [17]. Understanding the mechanisms underlying the development of prostate cancer and the contribution of fusion gene products is crucial to reduce cancer mortality in AA men.

The primary objective of this study was to examine molecular differences in fusion genes in a patient cohort that we examined previously for differences in gene expression patterns. This initial study supported the concept that inflammatory processes may contribute to disease progression in AA men [18]. Here, we undertook an additional series of additional genome-wide expression profiling experiments using high throughput (HT) RNA sequencing coupled with fusion gene prediction computational approaches to assess and compare fusion gene burden in AA men relative to EA. Our research provides novelty in the context of (1) the identification of novel putative fusions in prostate tumor tissue and (2) exploiting novel bioinformatics methods to predict fusions in the context of study on racial diversity.

## 2. Materials and Methods

### 2.1. Human Subjects

The study was authorized by the Institutional Review Board (IRB) of the Medical University of South Carolina (MUSC) and the Ralph H. Johnson VA Medical Center (VAMC), and by the Research and Development (R&D) Committee of the VAMC and performed as an interventional study of a new drug (IND) 77839, approved by the Food and Drug Administration (FDA). The 27 male subjects (10 AA and 17 EA men) enrolled in the study were all diagnosed with localized prostate cancer and had selected surgical removal of the prostate (prostatectomy) as a treatment. The current standard of care requires a two-month interval between biopsy and prostatectomy to reduce inflammation caused by the biopsy. Enrolled subjects were randomized to vitamin D3 supplementation at 4000 IU per day or placebo for two months prior to surgery. Two blood samples were obtained from each patient (at enrollment and on the day of the prostatectomy) to assay serum levels of 25-hydroxyvitamin D3 [25(OH)D3]. Fourteen subjects (5 AA and 9 EA) took 4000 international units (IU) of vitamin D3 per day for two months prior to surgery; 13 subjects (5 AA and 8 EA men) received placebo for two months prior to surgery. Based on the serum levels of 25(OH)D at study exit, we concluded that there was a high level of compliance by all enrolled subjects [18]. All 14 subjects that received vitamin D3 supplementation had an increase in their serum concentration of 25(OH)D3 [18]. Table 1 provides an overview of the patient characteristics and clinical information. A more detailed description of the patient clinical data and demographic background is provided in Supplemental Table S1.

### 2.2. Tissue Sample Procurement and RNA Purification

Surgical specimens were received in the frozen section laboratory at the Medical University of South Carolina or the Ralph H. Johnson VA Medical Center. Nonmalignant tissue samples were removed from the peripheral zone of the prostate under the supervision of the attending pathologist to ensure that the excision of tissue samples did not interfere with the diagnostic priorities. Tissue samples were transferred to sterile tubes, quick-frozen in liquid nitrogen, and transported to the Hollings Cancer Center Genomics Core Facility. RNA from each de-identified tissue sample was extracted using Qiagen RNeasy. RNA integrity was assessed using an Agilent 2100 Bioanalyzer and RNA 6000 Nano Assay chip (Agilent Technologies, Palo Alto, CA, USA).

### 2.3. RNA Sequencing (RNA-seq) and Quality Control

**Figure 1:**
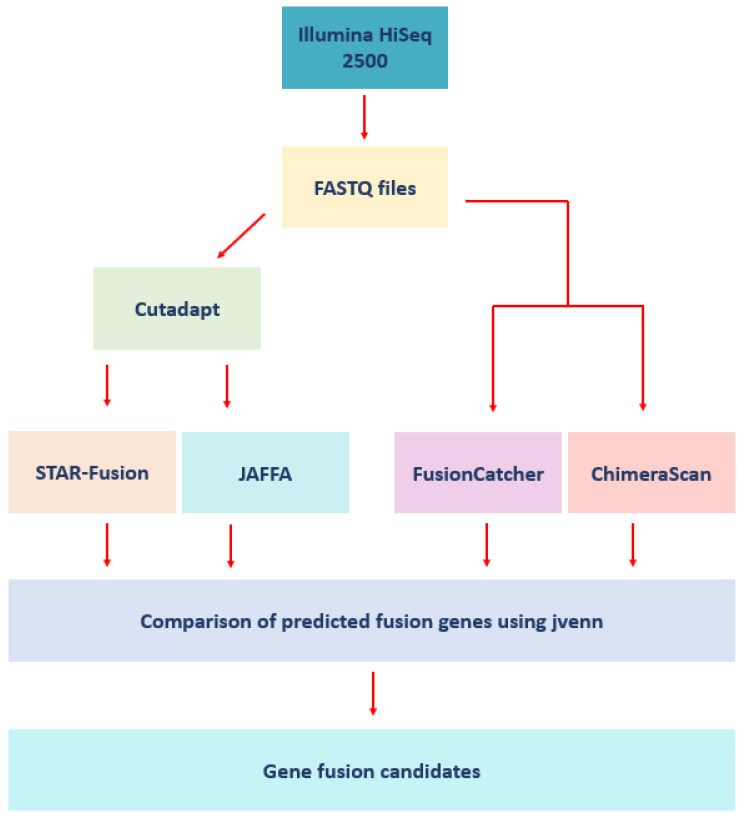
Workflow schematic. Fastq files were inputted to FastQC and Cutadapt. Four separate gene fusion tools predicted gene fusions that were compared using jvenn.

A 100–200 ng total RNA sample was used to prepare RNA-seq libraries using the TruSeq RNA Sample Prep Kit (Illumina, San Diego, CA, USA). High throughput sequencing (HTS) was performed using an Illumina HiSeq 2500 with each sample sequenced to an average depth of >60 million reads using a mix of paired-end (PE125) and single-end (SE50) sequencing strategies. All raw sequencing data were submitted to the NCBI GEO database (accession GSE152929). Quality control (QC) was performed using Cutadapt (v1.18) [19]. Low-quality and over-represented sequences identified by FastQC were removed [20]. Cutadapt processed data were used as inputs for fusion detection analysis unless raw data were required by the fusion prediction tool (Figure 1). A total of 2,052,016,465 reads across all 27 patients were inputted into this analytical pipeline. 

### 2.4. Fusion Detection Software

Four fusion detection tools were used to identify fusion genes in the 27 samples, STAR-Fusion (v1.7.0), FusionCatcher (v1.20) [21], JAFFA (v1.09) [22], and ChimeraScan (v0.4.5), using a mix of single (SE) and/or paired-end (PE) reads (Figure 1) [23]. These tools were selected based on availability and support at the time of analysis and the prior comparative assessment of methods for fusion transcripts detection from RNA-seq data by Kumar and colleagues [24]. These software packages are freely available to the scientific community and have been trained, assessed, and benchmarked on diverse types of datasets, and utilize distinct algorithms. 

### 2.5. Benchmarking the Fusion Detection Tools Using Prostate Cancer Cell Lines

In order to benchmark and validate the fusion analysis tools for PC analyses, we selected PC cell lines that were known to be positive or negative for the presence of the *TMPRSS2:ERG* fusion. A summary of the cell lines examined is provided in Supplemental Table S2. We determined whether each tool could detect this fusion or not. The cell lines examined included VCaP (+), HCIH-660 (+), PC3 (−), LNCaP (−), DU145 (−) and 22RV1 (−), PrECLH (unknown), and MDA-PCa-2B (unknown) for *TMPRSS2:ERG* status. Sequence data were downloaded from the NCBI Sequence Read Archive (accession code PRJNA523380). This provided a series of positive and negative controls for the detection of *TMPRSS2:ERG* and allowed determination of the accuracy of the fusion prediction tools in the context of prostate cancer. HCIH-660 and VCAP both served as positive controls, and PC3, LNCaP, DU145, and 22RV1 as negative controls. All cell line RNA-seq data were available in paired-end format. The cell lines were analyzed using STAR-Fusion, FusionCatcher, and JAFFA with the software versions, reference genome, and tool parameters described in Section 2.4.

### 2.6. Analysis of the Patient Samples

Our patient samples were analyzed for the presence of fusion genes using STAR-Fusion, FusionCatcher, JAFFA, and ChimeraScan. ChimeraScan analyses PE data only, which resulted in 17 samples analyzed with this tool. STAR-Fusion and FusionCatcher can analyze SE reads; however, they require a minimum input of 100 and 130 bp, respectively. STAR-Fusion analyzed all our SE reads; however, no fusions were predicted. Analysis of the PE samples with STAR-Fusion (7 EA and 10 AA) detected fusion genes. FusionCatcher failed to analyze the SE samples, again limiting the results to 17 samples. Table 2 provides a summary of the input data used for each fusion detection tool. Fusion plots were created by Chimeraviz v1.19.0 using the R statistical environment [25].

Inputs for these fusion prediction tools follow: FusionCatcher and ChimeraScan (raw fastq files) and STAR-Fusion and JAFFA (preprocessed fastq files). Genome build GRCh38 was used as the reference for STAR-Fusion, FusionCatcher, and JAFFA, while ChimeraScan employed GRCh37. Default tool parameters were utilized for STAR-Fusion and FusionCatcher. For JAFFA, the direct mode was used, and the fusion predictions were classified as “high”, “medium”, or “low” confidence. Fusion genes with low-confidence ratings were excluded from downstream analyses. Shared predicted fusion genes between samples were investigated using jvenn [26]. The salient features of the fusion detection tools are summarized in Table 3. 

## 3. Results

### 3.1. Cell Line Analysis

*TMPRSS2:ERG* was predicted in both positive control cell lines (VCaP and HCIH-660) using STAR-Fusion, FusionCatcher, and JAFFA. JAFFA predicted this fusion with high confidence in both VCAP and HCIH-660. All tools analyzing the negative control cell lines did not predict the presence of *TMPRSS2-ERG*. Supplementary Table S3 provides an unfiltered list of the fusion genes predicted by each tool and organized by cell line.

### 3.2. STAR-Fusion

STAR-Fusion identified 34 fusion genes in total with duplicates removed. The tool predicted fusions from PE reads (10 AA, 7 EA). No fusions were obtained with the SE data. Of these 17 patients for which there was PE data, 10 AA patients yielded 26 predicted fusion genes compared to only 10 fusions obtained from the 5 EA patients (with 2 common fusions shared between AA and EA patients). Two of the EA patients did not reveal any fusion genes. Supplementary Table S4 provides the unfiltered list of the fusion genes predicted by STAR-Fusion. 

### 3.3. FusionCatcher

FusionCatcher predicted 4918 fusion genes across 17 patients with duplicates removed. FusionCatcher enables the analysis of SE reads; however, they must be ≥130 bp. The SE reads were thus too short and therefore not compatible with FusionCatcher, decreasing the total patient count examined to 17 patients. Ten AA patients yielded 4753 fusion genes compared to only 278 for the 7 EA patients examined (with 113 common fusions shared between AA and EA patients). Supplementary Table S4 lists all unfiltered fusion genes predicted by FusionCatcher. 

### 3.4. JAFFA

JAFFA was run using the “direct” mode option, and exploits sequence reads that do not map to known transcripts. In total, JAFFA predicted 4772 high- and medium-confidence fusion genes across all 27 patients with fusion duplicates removed. AA patients accounted for 3290 of these predicted fusion genes compared to 1530 fusion genes in EA patients (with 48 shared fusions between AA and EA patients). Supplementary Table S4 provides an unfiltered list of all fusion genes predicted by JAFFA. 

### 3.5. ChimeraScan

ChimeraScan predicted a total of 1621 fusion genes across 17 patients (duplicates removed). SE reads are not compatible with ChimeraScan; 1134 fusions were detected in AA patients and 991 patients of European descent (504 common fusions between AA and EA patients). Supplementary Table S4 provides a complete original list of the fusion genes predicted by ChimeraScan. 

### 3.6. Comparison of Fusion Genes Based on Race

**Figure 2:**
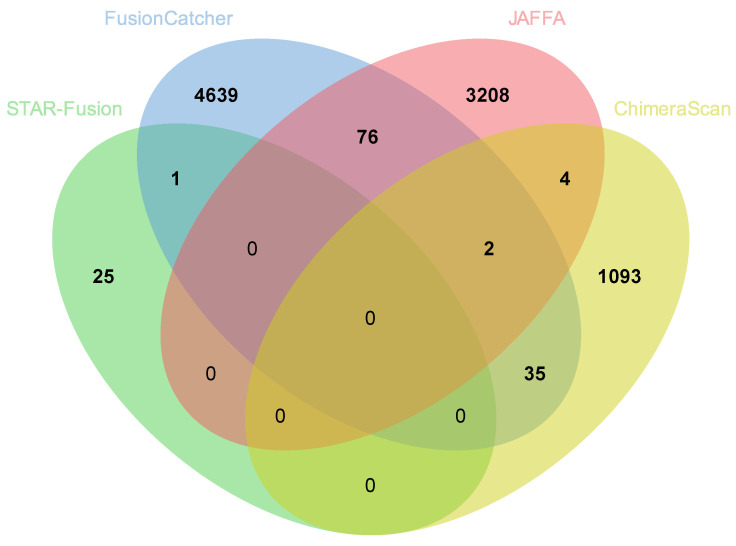
Comparison of fusion genes predicted by the different pipelines in African American patients. Total nonredundant fusions = 9203.

**Figure 3:**
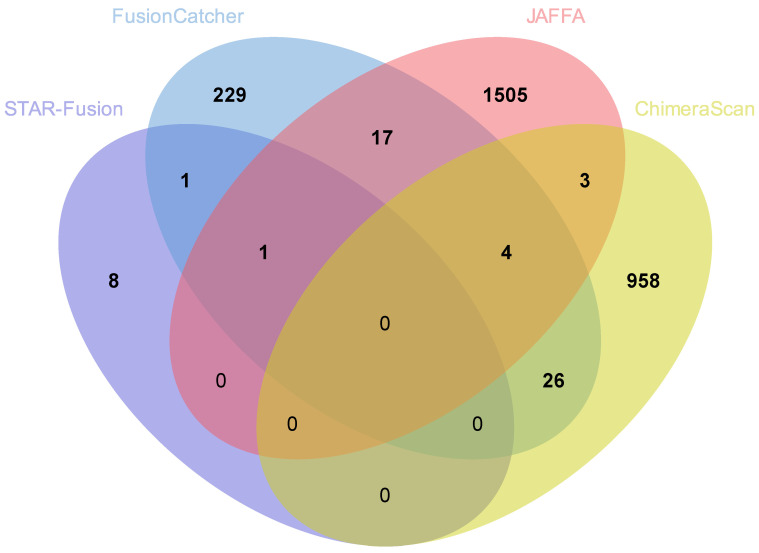
Comparison of fusion genes predicted by the different pipelines in European American patients. Total nonredundant fusions = 2809.

**Figure 4:**
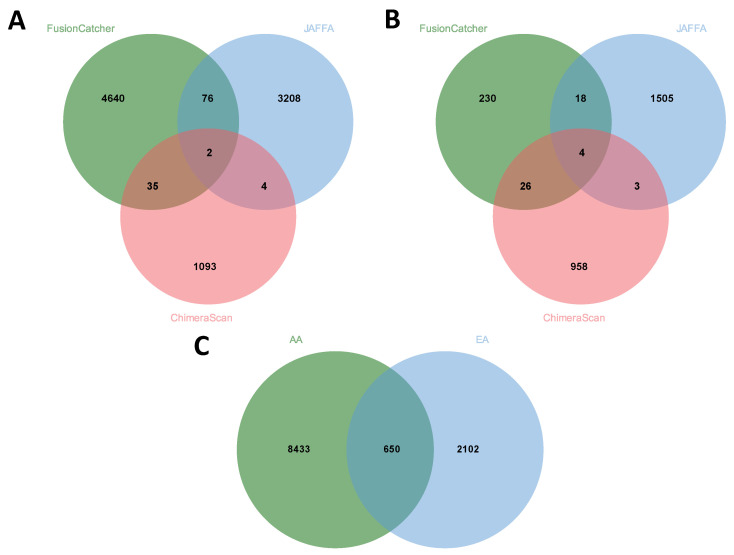
(**A**) Comparison of fusions detected by FusionCatcher, JAFFA, and ChimeraScan in AA patients. Two shared fusion genes were predicted by these tools in AA patients (*NAIP:OCLN* and *PDE1C:DNAJC6*). (**B**) Comparison of fusions detected by FusionCatcher, JAFFA, and ChimeraScan in EA patients. Four shared fusion genes were predicted by these tools in EA patients (Shared fusions—*NAIP:OCLN*, *FOXP2:CREM*, *KANSL1:ARL17B*, and *KANSL1:ARL17A*). (**C**) Comparison of detected fusions in AA and EA patients across all four tools. Low-confidence fusions have been excluded from all the comparisons.

The total number of fusion genes predicted in AA patients was more than three times (9083) that predicted in EA (2752) based on a summation of fusions predicted by all four tools with duplicates removed. Comparisons of all fusions based on STAR-Fusion, FusionCatcher, JAFFA, and ChimeraScan and across AA and EA are provided in Figure 2 (AA) and Figure 3 (EA), respectively. Comparisons excluding STAR-Fusion, which generated fewer predicted fusions, are presented in Figure 4A,B. Table S4 contains unfiltered data outputted by all fusion detection tools. The fusion counts we report are based on a summation of all fusions with duplicated fusions removed. Duplicated fusions between races in each tool are kept in the table and are included in total fusion count.

No single fusion was detected by all four programs, reflecting differences in the underlying algorithms for how each tool predicts fusion genes [24] (Figure 2 and Figure 3; Table 2 and Table 3). Table 4 summarizes fusion genes detected with each tool and these were organized by race to enable comparisons. Of the 12,012 fusions genes predicted across all four tools, 8433 were unique to AA patients and 2102 unique to EA patients. Only 650 predicted fusions were shared between AA and EA (Figure 4C). 

An expanded description of the fusions and the output data from the tools are provided in Supplemental Table S4. A summary of novel predicted fusion genes is presented in Supplemental Table S5. Racial differences in predicted fusion genes are summarized in Supplemental Table S6. Shared fusions across EA and AA patients are presented in Supplemental Tables S7 and S8, respectively.

Novel fusion genes were detected with all four programs (Table 5 and Supplemental Table S5). *NAIP:OCLN* was identified as a novel fusion in AA patients (patient IDs AA 19, AA 23, and AA 27; Supplemental Table S1). This fusion gene was also observed in one EA patient (patient ID EA 3; Supplemental Table S1). *PDE1C:DNAJC6* was detected as a novel fusion gene in one AA patient (patient ID AA 19; Supplemental Table S1). The fusion gene *KANSL1:ARL17B* was predicted in EA patients IDs EA 3, EA 4, and EA 5. *AHSA1:DLG3* was detected in one EA patient (patient ID EA 3; Supplemental Table S1) and finally *FOXP2:CREM* was detected in one EA patient (ID EA 1) by FusionCatcher, JAFFA, and ChimeraScan. These chimeras are candidates for further study as they were identified as predicted fusions using different tools, which suggests that they have biological relevance. Of particular interest are the fusions *NAIP:OCLN* and *KANSL1:ARL17B*, which were predicted in AA and EA patients multiple times.

All shared fusion genes were cross-checked with existing fusion databases including the Mitelman Database of Chromosome Aberrations [27] to determine if they had been previously associated with cancer. Additionally, these fusions were assessed using the TumorFusions data portal (http://tumorfusions.org/) (accessed on 27 August 2021) [28] based on the PRADA (Pipeline for RNA-Sequencing Data Analysis) pipeline and prostate adenocarcinoma data (PRAD) from TCGA to determine if they had been previously reported in prostate cancer [29]. From these database interrogations, all these predicted fusions are novel in the context of prostate cancer. 

**Figure 5:**
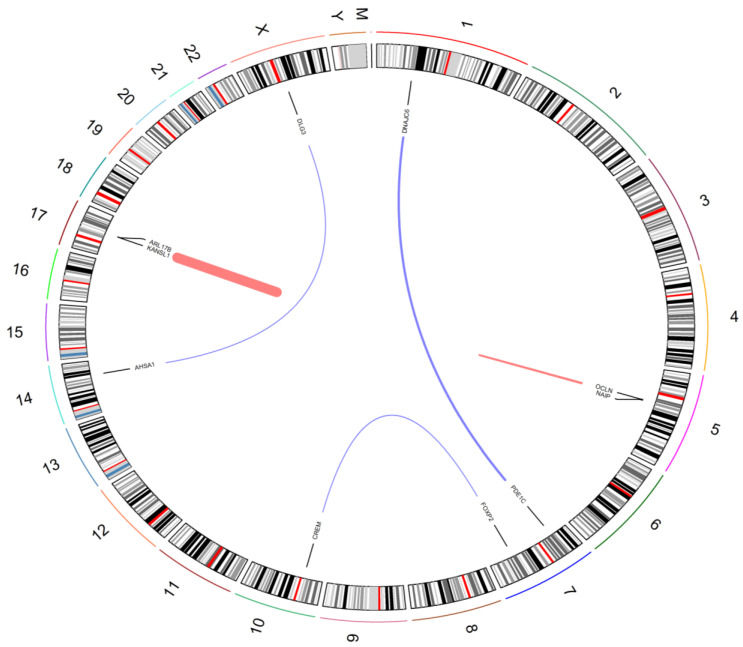
Chromosome Circos plot of novel fusion genes detected between fusion prediction tools. Red and blue lines indicate intra- and inter-chromosomal fusions. All chromosomes and cytoband information are denoted. The width of each link varies according to number of reads supporting the fusion event.

To visualize the novel predicted fusion genes we utilized Chimeraviz to generate a Circos plot (Figure 5). This provides a schematic of the different chromosomal regions that are combined together in the predicted fusion genes [25]. 

**Figure 6:**
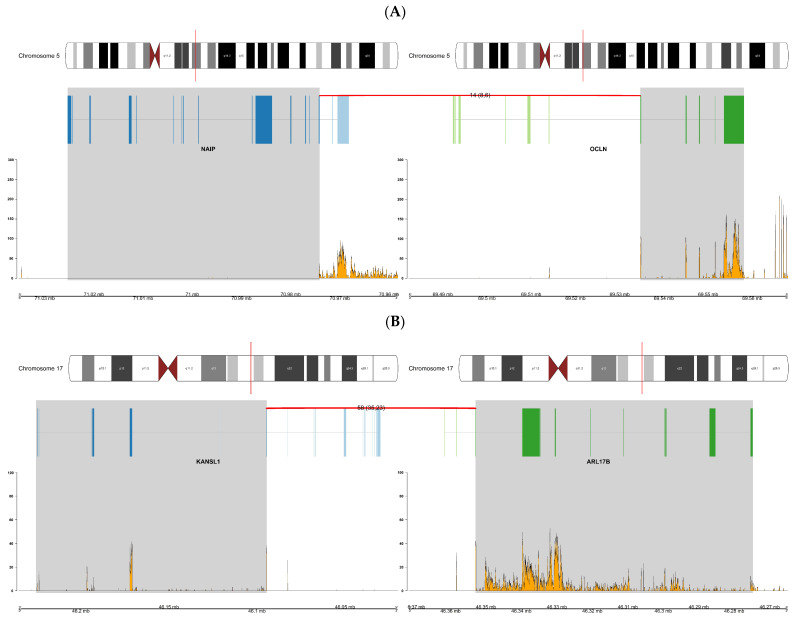
(**A**) Overview of the *NAIP:OCLN* fusion gene found in patient AA 20. The plot displays the locations on chromosome 5 of the fusion partner genes. (**B**) Overview of fusion *KANSL1:ARL17B* for patient EA 5. The plot displays the locations on chromosome 17 of the fusion partner genes. The plots display the locations on chromosome 5 and 17, respectively, of the fusion partner genes, the number of discordant (split and spanning, respectively, in parentheses) reads supporting the breakpoint indicated by the red line between partner genes, transcript information showing the exons in each partner gene, and, lastly, the RNA expression of partner genes beside their genomic location in Mb.

To explore the novel fusions more deeply, we generated a series of fusion plots. These plots present chromosome ideograms with a red line denoting the location of the two partner genes, along with transcript and breakpoint information. Figure 6 presents overviews of the novel fusions *NAIP:OCLN* (patient AA 20) and *KANSL1:ARL17B* (patient EA 5), which were the fusion genes most commonly predicted by fusion detection tools in patients of African and European descent. Data generated by FusionCatcher were used for creating all fusion plots as FusionCatcher was the tool that scored in the median range in terms of prediction performance (i.e., a good balance between specificity and sensitivity). The remaining plots for the fusions in Table 5 are presented as figures in the Supplemental Materials (Figures S1–S8).

## 4. Discussion

Chromosomal rearrangements resulting in gene fusions are the most common genetic alteration observed in cancer and our understanding of their relevance and function has significantly grown since the introduction of next-generation sequencing [13,30]. Fusions are defined as hybrid genes formed from merging component parts of two or more original genes [31]. They have been linked with malignancy and drive the progression of a wide range of cancers [31]. Fusion genes account for approximately 20% of cancer morbidity [32]. However, this rate significantly differs between cancers with many fusion genes specific to subtypes of cancer [32]. This makes them suitable molecular targets for cancer therapeutics because of their high specificity to cancerous cells [31]. Fusions are formed by genetic abnormalities, the most prevalent of which is chromosomal rearrangements that lead to the formation of novel fusion genes caused by chromosomal translocation, deletion, tandem duplications, or inversions [3,10,11,33]. This results in a hybrid product forming from the two individual genes [11]. Originally, fusion genes were thought to be only formed through chromosomal rearrangements; however, emerging evidence has suggested that chimeric RNAs can imitate gene fusions when the analogous gene fusion is absent [34]. Chimeras are formed from abnormal transcription caused by *trans*- and *cis*-splicing of pre-mRNAs, which then guide genome rearrangement to form the corresponding gene fusion [34,35]. This discovery has opened a source for novel biomarker and therapeutic target discovery efforts [35].

Cancer data from AA account for less than 12% of the Cancer Genome Atlas Portal (TCGA) compared to 77% for EA [36]. In the USA, significant and persistent racial disparities in prostate cancer outcomes persist. Prostate cancer disproportionately affects AA men in terms of incidence, morbidity, and mortality, even after adjustment for stage. AA men have an elevated risk of developing prostate cancer and a higher mortality rate compared to EA men. Although reduced access to and disconnect with the healthcare system contribute to racial disparities in prostate cancer outcomes, underlying genetic factors intensify these disparities. Thus, an understanding of the underlying biologic mechanisms is required to develop strategies to overcome them [37,38,39]. This also highlights the need for large-scale genomic and computational studies that encompass under-represented ethnic minority groups [11,40]. 

The objective of this study was to undertake a computational biology approach to uncover fusion genes in a cohort of patients that was previously assessed for differences in gene expression patterns between AA and EA men [18]. The patient cohort consisted of 10 AA and 17 EA men that underwent a prostatectomy. Fourteen of these subjects received vitamin D3 supplementation (4000 IU/day) and 13 subjects received placebo for two months before the surgery. Systems-level analysis indicated that AA men exhibited higher expression of genes associated with immune response and inflammation. 

Several fusion prediction tools were evaluated and considered before a final decision was made on which were most appropriate for study of our patients. The selection criteria involved the date the tool was developed, acceptance of the tool by the research community based on the number of times the software was cited, whether the software was maintained currently, and ease of installation. Programs that were considered but not used in this study included SOAPfusion [41], INTEGRATE [42], EricScript [43], and FusionBloom [44]. SOAPfusion uses the SOAPfusion aligner, which is reported to accurately discover fusion genes to a high sensitivity [41]. However, upon assessment we found that the software appears deprecated at this point. INTEGRATE is a relatively recent tool that has high sensitivity and precision compared to many other detection programs, making it an appealing choice [42]. However, it required the whole genome sequence of each patient, which was not available for this study. EricScript uses a combination of four alignment processes and is successful at being able to distinguish between true fusions and false positives and detecting chimeric fusions to a resolution of 1 bp. Although it exploits the transcriptome as a reference to reduce computational times [43], it also uses as input only paired-end data, and given the heterogeneity of our patient data, with some only available as SE reads, use of this tool was challenging. FusionBloom also appeared promising owing to its recent publication and availability as UNIX Make software. FusionBloom performed favorably when compared to STAR-Fusion with a low false positive rate for fusion predictions. However, after inspection it required many dependencies and ease of install was one of our criteria for program selection [44]. In summary, fusion gene prediction is a rapidly evolving field with tools quickly becoming obsolete or requiring many software dependencies that can become obsolete or generate IT security risks. Consequently, the choice of programs best suited to analysis of this nature will continue to evolve. 

The best known fusion genes associated with prostate cancer involve ETS transcription factor family members and the androgen-regulated promotor *TMPRSS2* (*TMPRSS2:ERG, TMPRSS2:ETV1,* and *TMPRSS2:ETV4*) [45]. The ETS-related gene (*ERG*) is the most common fusion partner for TMPRSS2 with *ETV1* and *ETV4* only ever reported in a small number of cases [46]. *TMPRSS2*:*ERG* fusions are a common genomic alteration in prostate cancer; however, variations in its incidence have been extensively observed. An 8% to 83% prevalence rate has been documented in the literature. These wide variations are explained by the origin of the tissue, laboratory techniques, and, of most interest, ethnic diversity [47]. The frequency of *TMPRSS2:ERG* fusions differ considerably by race. Men of European descent are typically more likely to harbor this fusion in comparison to men of African or Asian descent. A previous meta-analysis evaluating the prevalence of *TMPRSS2:ERG* fusions by race identified that only 25% of men of African descent compared to 49% for men of European ancestry had this fusion [47]. The literature suggests that *TMPSS2-ERG* fusions are inversely correlated with more aggressive forms of PC [48]. Biological pathways perturbed by this fusion are promising in the context of therapeutic target development; however, as this fusion is biased toward men of European descent, this suggests that other biological mechanisms are driving racial differences in PC [49]. 

Surprisingly, the *TMPRSS2:ERG* was not observed in any of our patient samples. We utilized well-established prostate cancer cell-line RNA-seq data to provide both positive and negative controls for benchmarking the fusion software. The cell lines VCaP and NCHI-660 are well known to harbor the *TMPRSS2:ERG* rearrangement. PC3, LNCaP, DU145, and 22RV1 are negative for *TMPRSS2:ERG* [50,51]. Research is limiting for PrECLH and MDA-PCa-2B and prior to our study it was unknown if these cells harbor *TMPRSS2:ETS*. We determined that they were negative for *TMPRSS2:ETS.* With *TMPRSS2:ERG* correctly predicted in both positive *TMPRSS2:ERG* and negative *TMPRSS2:ERG* cell lines with three of the fusion programs used in our pipeline, we conclude that *TMPRSS2:ERG* is absent in our patient cohort. There is a wide range of documented *TMPRSS2:ERG* prevalence recorded in literature, from ~8 to 83% [47]. The general consensus is that *TMPRSS2:ERG* is more common in patients of European ancestry. In our cohort, we did not see it as more prominent in our EA patients compared to AA patients.

Fusion genes as therapeutic targets in cancer treatment have been highly successful [32]. Chronic myelogenous leukemia is characterized by presence of the *BCR:ABL1* fusion and treated using imatinib mesylate (Gleevec) [52]. Our study reports several novel fusion gene transcripts uncovered using four separate fusion gene discovery tools. Each of the tools employs slightly different genome aligners and/or reference builds to identify new fusion genes, resulting in different candidates being predicted by each program, as well as variations in the numbers of overall fusion genes uncovered (Table 3). 

STAR-Fusion exploits several genomic resources that are available as a downloadable Genome Resource Lib. STAR the alignment tool aligns reads to a selected genome build, and captures spilt and discordant aligned reads. To identify candidate gene pairs representing potential gene fusions, identified discordant and split reads are mapped to exons of reference transcript annotations. Lastly, STAR-Fusion completes a series of filtering steps to predict the most likely true fusions. Filtering criteria include the strength of alignment, breakpoint proximity, and sequence similarity [53].

FusionCatcher completes several preprocessing quality steps before predicting fusions. This eliminates the need to complete adapter trimming using software such as Cutadapt or Trimmomatic. Preprocessing steps include: removing reads that contain adapters and poly-A/C/G/T tails; trimming reads based on quality scores; eliminating reads that align to ribosomal/transfer RNA, mitochondrial DNA, HLA genes and known viruses/phases/bacterial genomes; and, finally, removing reads that were identified as low quality by the Illumina sequencer [21]. FusionCatcher uses a combination of four aligners (STAR, Bowtie, Bowtie2, and BLAT) individually to predict fusion genes. Fusion genes that are predicted by all four methods are classified as candidate fusion genes [21].

ChimeraScan operates using Bowtie to align reads to a combined genome–transcriptome reference. Reads that fail a primary alignment step are segmented and realigned to uncover discordant reads. ChimeraScan then clusters these discordant reads and generates a list of putative 5′-3′ transcript pairs. Next, ChimeraScan completes junction alignment with the identified putative chimeric junction sequences. ChimeraScan completes this by generating a new reference index from the putative chimeric junction sequences and then realigning nominated candidate junction-spanning reads to the new reference index. Finally, ChimeraScan completes filtering steps to eliminate false positive fusion genes [23].

JAFFA’s first step is to remove alignments that align to intronic or intergenic regions. Next, sequences are transformed into a common form (tumor sequences) comprising either the assembled contigs or the reads themselves. JAFFA then completes a series of fusion detection steps consisting of aligning the tumor sequences to a reference transcriptome, counting the number of reads that support the breakpoint, aligning candidate fusions to a human genome, and narrowing the fusions based on a set criterion. Fusions that meet all criteria are categorized as high-confidence fusions [22]. 

JAFFA and ChimeraScan uncovered the highest number of fusion genes. STAR-Fusion identified the least number of fusion genes within each patient as well as the least diverse gene fusions. This may be partly due to its ability to exclude fusion genes that contain sequence-similar gene pairs and promiscuous fusion partners, or it could indicate a reduced sensitivity and greater specificity of the program compared to the others, due to its rigorous filtering steps [53]. 

Kumar et al. benchmarked the tools we used (except STAR-Fusion) by using positive, negative, mixed, and test datasets, and ranked them based on percentage sensitivity, defined as the number of true positives relative to total fusions, as follows: JAFFA (88%) > FusionCatcher (66%) > Chimerascan (8%). Based on our experience using each of the tools using our data and the model cell lines, our ranking is slightly different. We ranked our tools based on their accuracy to detect the fusion *TMPRSS2:ERG* in each of the prostate cancer cell lines, together with ease of use and the total time required. We considered the specificity of PE and SE reads per tool. Regarding this, JAFFA was the most accessible for analyzing SE reads. FusionCatcher and STAR-Fusion could analyze SE reads; however, the reads must be greater than a certain length to provide enough analytical power to predict fusion genes. ChimeraScan is incompatible with SE reads. Optimization is still required to enhance fusion prediction tools. We initially evaluated each tool based on its ability to correctly predict *TMPRSS2:ERG* in positive- and negative-control cell lines. We thus ranked the tools as follows: 1—STAR-Fusion, 2—FusionCatcher, 3—JAFFA, and 4—ChimeraScan. All three tools (STAR-Fusion, FusionCatcher, JAFFA) correctly predicted *TMPRSS2:ERG* in positive and negative controls; however, STAR-Fusion significantly outperformed FusionCatcher and JAFFA in terms of sensitivity. STAR-Fusion also performed on average more efficiently. All programs output the names of the two genes involved in the fusion event together with the chromosomal locations. Of the programs utilized, JAFFA was the only one to provide a confidence level for the predicted gene fusions, allowing further filtering to be undertaken by removing low-confidence predictions. For fusions to be classified as high confidence, certain conditions must be met, including having one or more spanning reads and pairs, breakpoints aligning to exon boundaries, and evidence of a rearrangement genomic gap greater than 200 kb [22]. Both FusionCatcher and JAFFA utilize more than one alignment tool. This increased the accuracy of the alignment and fusion break-point detection and may explain why these programs detected more fusion genes than STAR-Fusion [54]. JAFFA was the only tool that was able to predict fusions using SE50 samples. STAR-Fusion and FusionCatcher analyze SE reads; however, the reads must be at least 100 and 130 bases long, respectively. Reads less than the recommended length impacted the ability to predict fusions. 

Of the gene fusions found with these fusion detection programs, a few stand out for future study, particularly those commonly detected with at least three of the four tools and those identified in multiple patients of the same race. Fusions were cross-checked with existing fusion databases including the Mitelman Database of Chromosome Aberrations and TumorFusions.org to determine if they are novel or have already been associated with cancer [27,28]. There were no previous descriptions for the fusions *NAIP:OCLN*, *PDE1C:DNAJC6, KANSL1:ARL17B*, *FOXP2:CREM*, and *AHSA1:DLG3* in the context of prostate cancer. *NAIP:OCLN* has been previously described as an *NAIP->OCLN* promoter swap and associated with ectopic rearrangements contributing to evolutionary/genomic instability in humans. [55,56].

Our results show that there were many more fusion genes predicted for AA men compared to the EA men, even with fewer AA patients in our cohort. This emphasizes the high prevalence of predicted fusion genes within men of African descent. FusionCatcher, JAFFA, and Chimerscan all outputted the fusion gene *NAIP:OCLN*. This fusion was shared in three AA patients (Patient IDs AA20, AA22, and AA24) and one EA patient (ID EA15) (Supplemental Table S5). NLR family apoptosis inhibitory protein (*NAIP*) is a gene that is part of a 500 kb inverted duplication located on chromosome 5q13. Chromosome 5 is one of the largest chromosomes, yet it has a low gene density due to the presence of a high number of noncoding gene regions. Chromosome 5 is susceptible to rearrangements, deletions, and duplications. Deletion of this chromosome is common in PC and correlates with more aggressive disease [57]. This genetic alteration has also been observed with poor prognosis in chronic myelogenous leukemia [58]. Additionally, 5q13 lesions have been identified as a molecular target for increased risk in lung cancer [59] and are a common anomaly in breast cancer patients harboring *BRCA1* mutations [60]. Significant upregulation of *NAIP* in response to androgen deprivation therapy has been identified, suggesting it may have the ability to enhance survival of PC following androgen deprivation therapy [61]. Occludin (*OCLN*) encodes a main component of tight junctions and is involved in several cellular functions, with dysregulation in its function leading to neurological disorders [62,63]. Both *NAIP* and *OCLN* are upregulated 7.9-fold and 4.3-fold, respectively, in AA relative to EA (q = ^1 x 10-6^) [18]. The fusion of *NAIP* and *OCLN* was described previously by Micci et al., in endometrial stroma sarcoma [64] and by Iwakawa et al., in lung cancer cells [65]; however, this study is the first to report this gene fusion in prostate cancer. 

*PDE1C:DNAJC6* was also detected by JAFFA, FusionCatcher, and ChimeraScan in one AA patient (ID 24) (Supplemental Table S5). This fusion has previously been detected in thyroid cancer [66]. DNAJ heat shock protein family (Hsp40) member C6 (*DNAJC6*) is involved in the regulation of molecular chaperone activity through the stimulation of ATPase activity and is associated with Parkinson’s disease [67]. Phosphodiesterase 1C (*PDE1C*) encodes an enzyme that regulates proliferation and migration of vascular smooth muscle cells [68]. The group of phosphodiesterases (PDEs) regulates cyclic AMP and cyclic GMP. Variants in PDE genes have been linked with prostate cancer predisposition and progression [69].

*AHSA1:DLG3* was uncovered using three of the programs: STAR-Fusion, FusionCatcher, and JAFFA, in one EA patient (ID 15). *AHSA1* (activator of HSP90ATPase activity 1) is a gene involved in cell growth and proliferation, and migration and invasion through the Wnt/beta-catenin signaling pathway. It acts as a regulator of HSP90, and its activation by AHSA1 influences the metabolism of androgen in prostate cancer [70,71]. *DLG3* (discs large MAGUK scaffold protein 3) is upregulated in breast and ovarian carcinomas and downregulated in esophageal and papillary thyroid carcinoma [72].

*MYH11:THBS1* was found in one AA patient (ID 18) using STAR-Fusion, FusionCatcher, and JAFFA (Supplemental Table S5). Thrombospondin 1 (*THBS1*) encodes a protein that is a subunit of a homotrimeric protein that mediates cell-to-cell and cell-to-matrix interactions [73]. The decreased expression of *THBS1* has previously been seen in some human tumors, and its expression can be regulated by the tumor suppressor gene p53 [74]. Myosin heavy chain 11 (*MYH11*) encodes smooth muscle myosin heavy chain 11 protein, involved in transporting materials between and within cells. 

*KANSL1:ARL17B* was predicted by FusionCatcher, JAFFA, and ChimeraScan in EA patients (ID EA5, EA6, and EA15) (Supplemental Table S5). *KAT8* regulatory NSL complex subunit 1(*KANSL1*) encodes a protein that regulates gene expression through the modification of chromatin, playing a major role in the development of many body tissues [75]. ADP-ribosylation factor-like GTPase 17B (*ARL17B*) is involved in protein trafficking by modulating vesicle budding in the Golgi apparatus [76]. The fusion of *KANSL1* and *ARL17B* has been seen in leukemia [77] and in normal karyotype chronic myelogenous leukemia by Wen et al. (2012), where it is referred to as *KIAA1267-ARL17* but has not previously been observed in prostate cancer [78]. *KANSL1:ARL17A* was found by FusionCatcher, JAFFA, and ChimeraScan. *ARL17A* (ADP-ribosylation factor-like GTPase 17A) and its fusion with *KANSL1* has previously been seen within a cohort of patients with relapsing malignancies and is predicted to predispose a patient to cancer as it is linked to histone acetylation, but it has not yet been associated directly with prostate cancer [79]. 

The *FOXP2:CREM* gene fusion was detected by FusionCatcher, JAFFA, and ChimeraScan in one EA patient (ID 1) (Supplemental Table S5). Forkhead box p2 (*FOXP2*) is involved in the production of a transcription factor protein that regulates the activity of other genes [80]. Its dysregulation is thought to be involved in cancer progression as its aberrant expression has been seen in various cancer types, either downregulated or upregulated [81]. CAMP responsive element modulator (*CREM*) is a gene that also encodes a transcription factor that helps to regulate the response to CAMP pathways, which can be mediators of cellular proliferation [82]. The fusion of *FOXP2* and *CREM* is novel and has not yet been associated with prostate cancer. 

## 5. Conclusions

The goal of this study was to exploit computational biology approaches to predict novel fusion genes and analyze these in the context of prostate cancer, especially as it relates to differences between EA and AA men. With this objective in mind, we interrogated the prostate transcriptome using prostate tissue specimens obtained at surgery from prostate cancer patients and assessed the samples for the presence of novel fusion genes using four different tools. We discovered that the predicted fusion gene burden is almost three times higher in AA patients compared to EA. A previous study investigating the burden of observed genomic alterations in the context of genetic ancestry (AA vs. EA) in PC observed the total number of identified fusion genes did not significantly differ based on ethnicity (*p* = 0.59) [17]. Thus, further predictive computational studies are required to characterize the prostate cancer fusion gene transcriptome. 

The underlying genetic elements behind cancer differ between patients, meaning their responses to generic treatments will vary. Precision medicine is an expanding field of cancer treatment and diagnosis, aiming to identify those at higher risk of cancer development [83]. The continued development of fusion gene detection pipelines has enabled an expansion in fusion gene discoveries with higher accuracy and shorter computing times. These fusion discoveries have the potential to improve cancer diagnosis, prognosis, and guide therapeutic development. As the fusions above were found by more than one of the computational approaches tested, it is likely that these are significant in the context of prostate cancer biology in AA patients. Future work will assess whether these gene fusions are drivers of cancer progression or passenger mutations. Gene ablation studies are required to assess whether these fusion genes in prostate cell lines of AA origin influence growth and development of prostate cells. 

The limitations of this study are the small sample size and the lack of germline genomic data from these individuals. Although the dataset was from a relatively small sample of patients, its high-quality, depth of sequencing, and representation of a patient group under-represented in genomic studies make it a unique resource and allowed us to develop a framework for fusion gene predictions. The fact that many of these fusions were not described previously from genome sequencing efforts suggests that these somatic fusion gene mutations may be relevant in the context of prostate cancer disparities. 

## Figures and Tables

**Table 1:** Patient demographics and clinical characteristics.

	European American (*n* = 17)	African American (*n* = 10)
**Age, years, mean (SD)**	61.4 (4.78)	63.4 (5.21)
**Radical Prostatectomy** **Gleason Score, *n* (%)**		
3 + 3	4 (23.5)	2 (20.0)
3 + 4	10 (58.8)	7 (70.0)
4 + 3	1 (5.88)	0 (0.00)
4 + 3 + 5	2 (11.8)	1 (10.0)
**Tumor, *n* (%)**		
Confined	15 (88.2)	7 (70.0)
Extension	2 (11.8)	3 (30.0)
**Nodal Involvement, *n* (%)**		
N0	13 (76.5)	7 (70.0)
NX	4 (23.5)	2 (20.0)

**Table 2:** Comparison of the input sequencing data, patient samples, and genome build specific to our analyses.

Program	STAR-Fusion	FusionCatcher	JAFFA	ChimeraScan
**Data type**	Single and paired end	Single and paired end	Single and paired end	Paired end only
**Preprocessing (Y/N)**	Y	N	Y	N
**Samples analyzed**	27 samples	17 samples	27 samples	17 samples
	10 AA	10 AA	10 AA	10 AA
	17 EA	7 EA	17 EA	7 EA
**Genome build**	GRCh38	GRCh38	GRCh38	GRCh37

**Table 3:** Fusion detection pipelines.

Program	STAR-Fusion	FusionCatcher	JAFFA	ChimeraScan
**Release year**	2017	2012	2015	2011
**Installation**		Python script	Java script	C++ script
**Alignment tools**	STAR	Bowtie, Bowtie2, Liftover, STAR, Velvet, FaToTwoBit, SAMtools, Seqtk, Numpy, Biopython, Picard, Parallel	Bpipe, Velvet, Oases, SAMtools, Bowtie2, BLAT, Dedupe, Reformat	Bowtie
**Reference**	Transcriptome	Genome and transcriptome	Transcriptome	Genome and transcriptome
**Reads format**	Single end (suggested reads > 100 bp) and paired end	Single end (reads > 130 bp) and paired end	Single end (any length) and paired end	Paired end
**Class**	Read mapping	Read mapping	Read mapping	Read mapping
**Input data**	RNA-Seq	RNA-Seq	RNA-Seq	RNA-Seq
**Human Assembly**	GRCh38	GRCh38	GRCh38	GRCh37

**Table 4:** Comparison of fusion genes predicted by the four detection tools. (Low-confidence fusions detected by JAFFA were excluded from this comparison).

Number of Different Fusion Genes Detected by Each Tool					
Group	STAR-Fusion	FusionCatcher	JAFFA	ChimeraScan	Total (Nonredundant) Fusions
**AA**	26	4753	3290	1134	9203
**EA**	10	278	1530	991	2809
**Total**	36	5031	4820	2125	

**Table 5:** Novel predicted fusion genes identified by each tool. Race, patient samples, and the tools used to obtain the fusions are summarized.

Fusion Gene	Race	Patient ID	Tools
*NAIP:OCLN*	AA	AA 20, 22, 24	ChimeraScan, FusionCatcher, JAFFA
*NAIP:OCLN*	EA	EA 15	ChimeraScan, FusionCatcher, JAFFA
*PDE1C:DNAJC6*	AA	AA 24	ChimeraScan, FusionCatcher, JAFFA
*KANSL1:ARL17B*	EA	EA 5, 6, 15	ChimeraScan, FusionCatcher, JAFFA
*FOXP2:CREM*	EA	EA 1	ChimeraScan, FusionCatcher, JAFFA
*AHSA1:DLG3*	EA	EA 15	STAR-Fusion, FusionCatcher, JAFFA

## Data Availability

The data that support the findings of this study are available at National Center for Biotechnology Information (NCBI) Gene Expression Omnibus (GEO) database; accession number GSE152929, https://www.ncbi.nlm.nih.gov/geo/query/acc.cgi?acc=GSE152929, accessed on 16 April 2022.

## Supplementary Materials

The following supporting information can be downloaded at: https://www.mdpi.com/article/10.3390/biology11050625/s1, Figure S1: Overview of fusion NAIP:OCLN for African American patient 22; Figure S2: Overview of fusion NAIP:OCLN for African American patient 24; Figure S3: Overview of fusion NAIP:OCLN for European American patient 15; Figure S4: Overview of fusion PDE1C:DNAJC6 for African American patient 24; Figure S5: Overview of fusion KANSL1:ARL17B for European American patient 6; Figure S6: Overview of fusion KANSL1:ARL17B for European American patient 15; Figure S7: Overview of fusion FOXP2:CREM for European American patient 1.; Figure S8: Overview of fusion FOXP2:CREM for European American patient 1.; Table S1: Patients IDs, demographic and clinical information; Table S2: Summary of model cell lines; Table S3: Cell line results organized by tool; Table S4: Predicted fusions detected by each of the four programs, STAR-Fusion, FusionCatcher, JAFFA and ChimeraScan respectively; Table S5: Summary of predicted fusion genes; Table S6: Racial differences in predicted fusion genes in in African American and European American men; Table S7: Shared fusions across EA patients using the fusion prediction tools.; Table S8: Shared fusions across AA patients using the fusion prediction tools.
