# Supplementary material for: Fusion Genes in Prostate Cancer: A Comparison in Men of African and European Descent
Source: Biology (Basel). 2022 Apr 20;11(5):625. doi: 10.3390/biology11050625 (PMC9137560; doi:10.3390/biology11050625)
Supplement: Supplementary file 1 [file biology-11-00625-s001.zip › supplemental_with_figures_02_04_22.pdf]

Supplemental Materials

Fusion genes in prostate cancer. A comparison in men of African and European descent.

Rebecca Morgan[1], Dulcie Keeley[1], E. Starr Hazard[2], Emma H. Allott[3], Bethany Wolf[4], Stephen J. Savage[5,6], Chanita Hughes Halbert [7,8,9.10], Sebastiano Gattoni-Celli[6],[11] and Gary Hardiman[1,4, 12]*

1 Faculty of Medicine, Health and Life Sciences, School of Biological Sciences & Institute for Global Food Security (IGFS) Queen's University Belfast (QUB), NI, UK; rmorgan21@qub.ac.uk, dkeeley02@qub.ac.uk, g.hardiman@qub.ac.uk

2 Academic Affairs Faculty, Medical University of South Carolina (MUSC), Charleston, SC 29425 hazardes3@gmail.com

3 Patrick G. Johnston Centre for Cancer Research, QUB, Belfast, Northern Ireland, UK E.Allott@qub.ac.uk

4 Department of Public Health Sciences, MUSC

5 Department of Urology, MUSC, Charleston, SC 29425 savages@musc.edu

6 Ralph H. Johnson VA Medical Center, Charleston, SC USA

7 Hollings Cancer Center, MUSC, Charleston, SC 29425 hughesha@musc.edu

8 Department of Psychiatry & Behavioral Sciences, MUSC, Charleston, SC 29425

9 Department of Population and Public Health Sciences, University of Southern California, Los Angeles, CA 90032

10 Norris Comprehensive Cancer Center, University of Southern California, Los Angeles, CA, 90033 hughesha@usc.edu

11 Department of Radiation Oncology, MUSC, Charleston, SC 29425 Sebastiano.Gattoni-Celli@bms.com

12 Department of Medicine, MUSC, Charleston, SC 29425

*Correspondence: (GH) G.Hardiman@qub.ac.uk

Figure Legends

Figure S1

Overview of fusion NAIP:OCLN for African American patient 22.

Figure S2

Overview of fusion NAIP:OCLN for African American patient 24.

Figure S3

Overview of fusion NAIP:OCLN for European American patient 15.

Figure S4

Overview of fusion PDE1C:DNAJC6 for African American patient 24.

Figure S5

Overview of fusion KANSL1:ARL17B for European American patient 6.

Figure S6

Overview of fusion KANSL1:ARL17B for European American patient 15.

Figure S7

Overview of fusion FOXP2:CREM for European American patient 1.

Figure S8

Overview of fusion AHSA1:DLG3 for European American patient 15.

Tables

Table S1

Patients IDs, demographic, and clinical information.

Table S2

Summary of model cell lines.

Table S3

Predicted fusions detected by STAR-Fusion, FusionCatcher and JAFFA using model cell lines.

Table S4

Unfiltered fusions detected by each of the four programs, STAR-Fusion, FusionCatcher, JAFFA and ChimeraScan respectively.

Table S5

Summary of predicted fusion genes.

Table S6

Racial differences in predicted fusion genes in in African American and European American men.

Table S7

Shared fusions across EA patients using the fusion prediction tools.

Table S8

Shared fusions across AA patients using the fusion prediction tool.

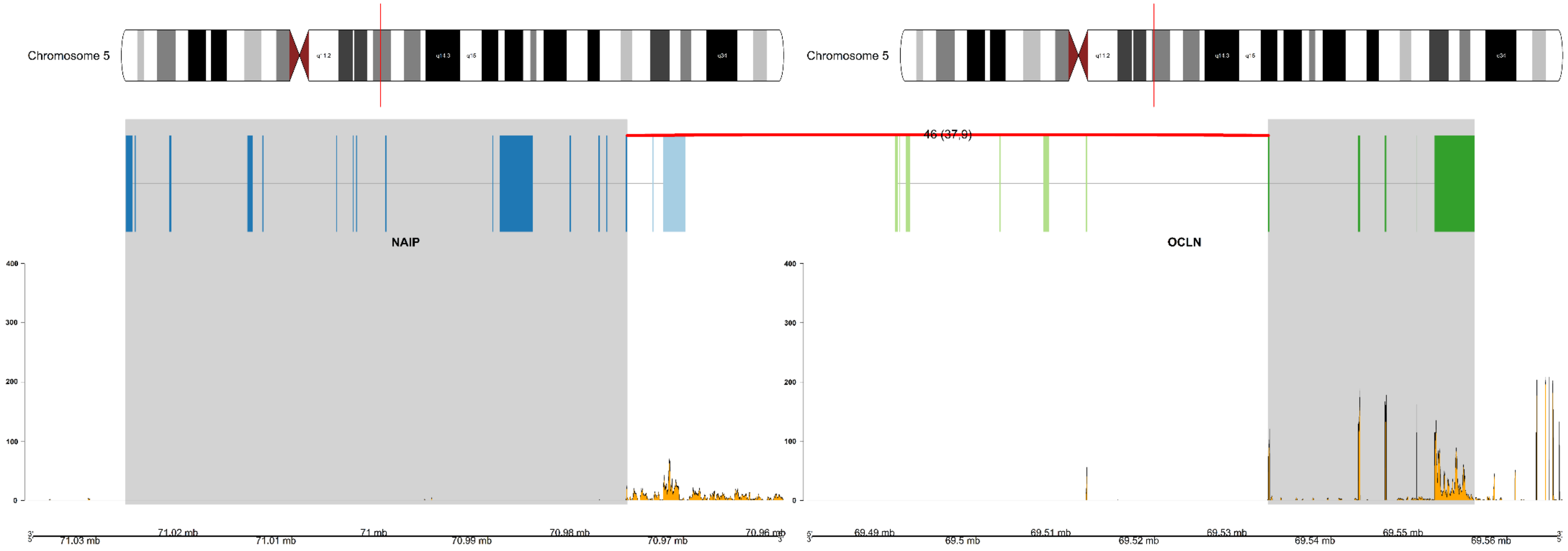

Figure S1

Overview of fusion NAIP:OCLN for African American patient 22.

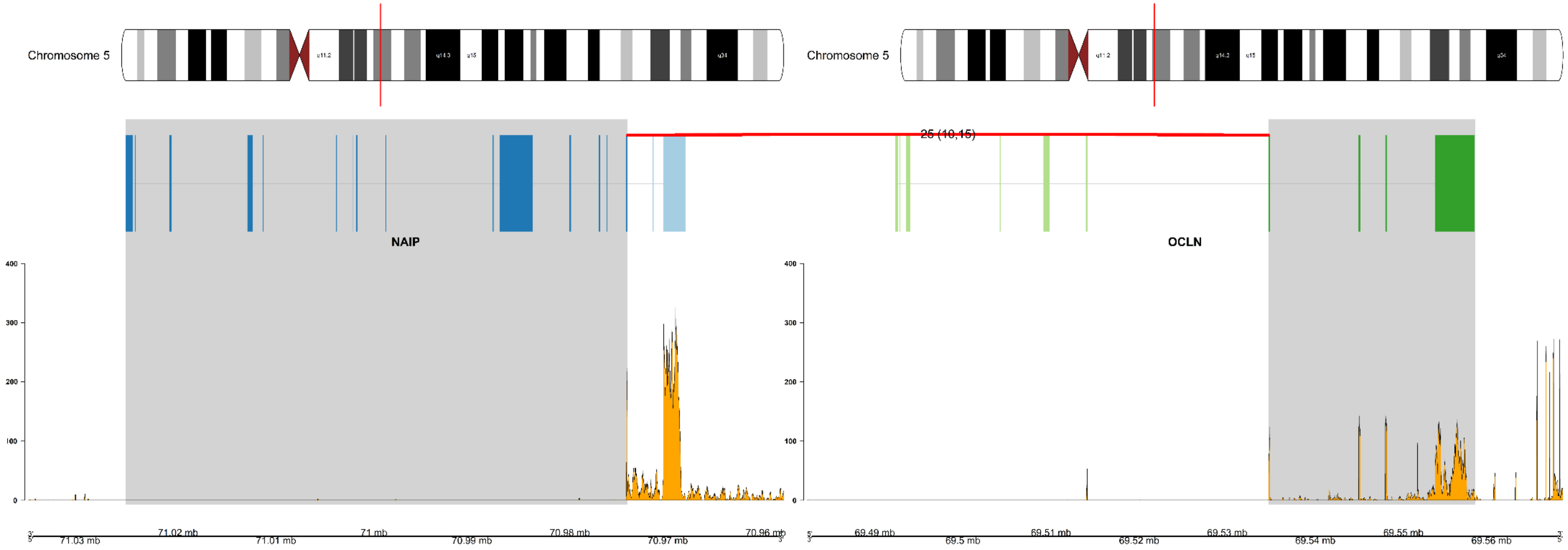

Figure S2

Overview of fusion NAIP:OCLN for African American patient 24.

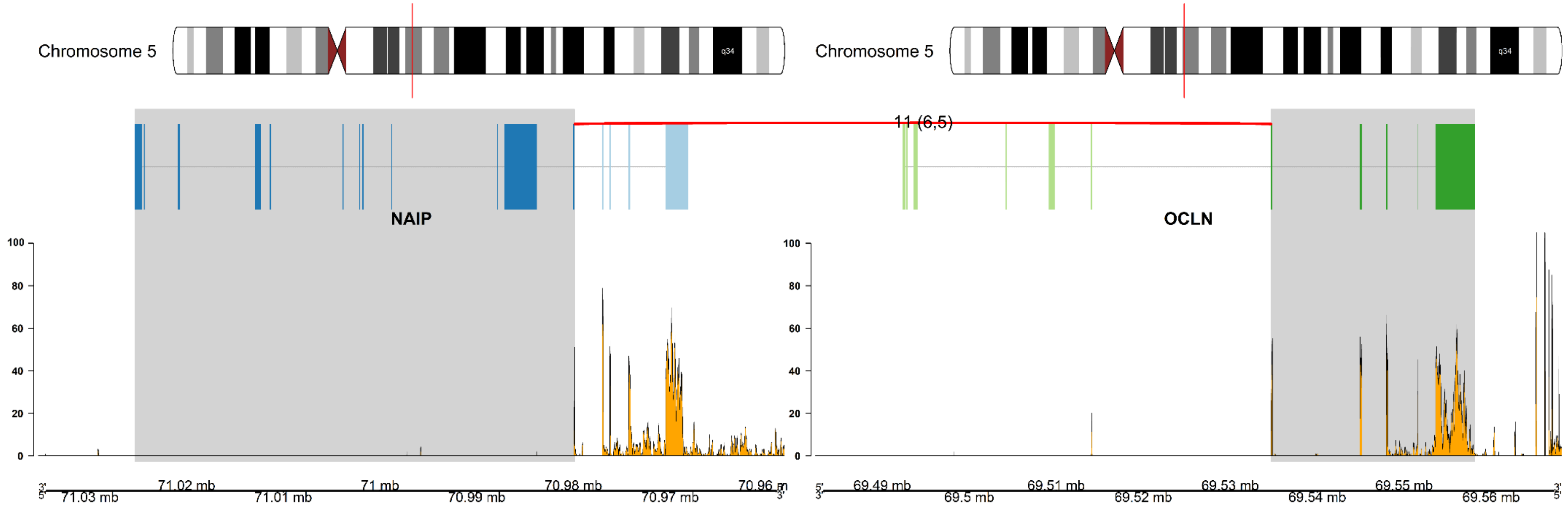

Figure S3

Overview of fusion NAIP:OCLN for European American patient 15.

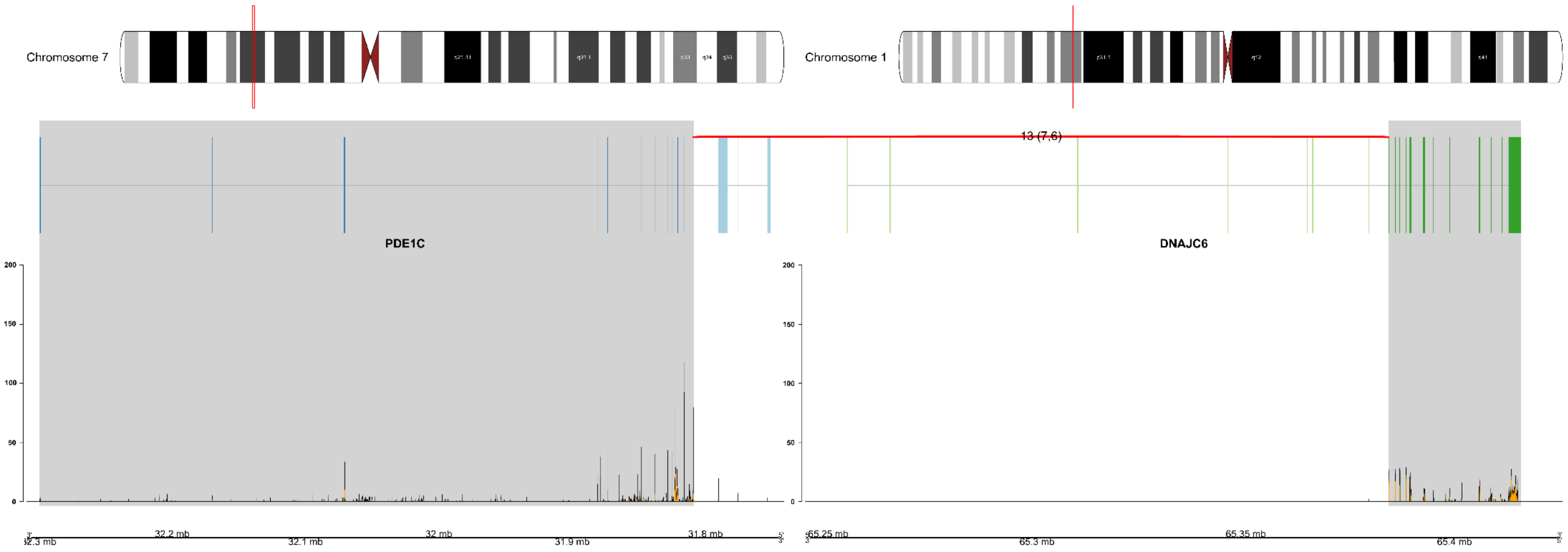

Figure S4

Overview of fusion PDE1C:DNAJC6 for African American patient 24.

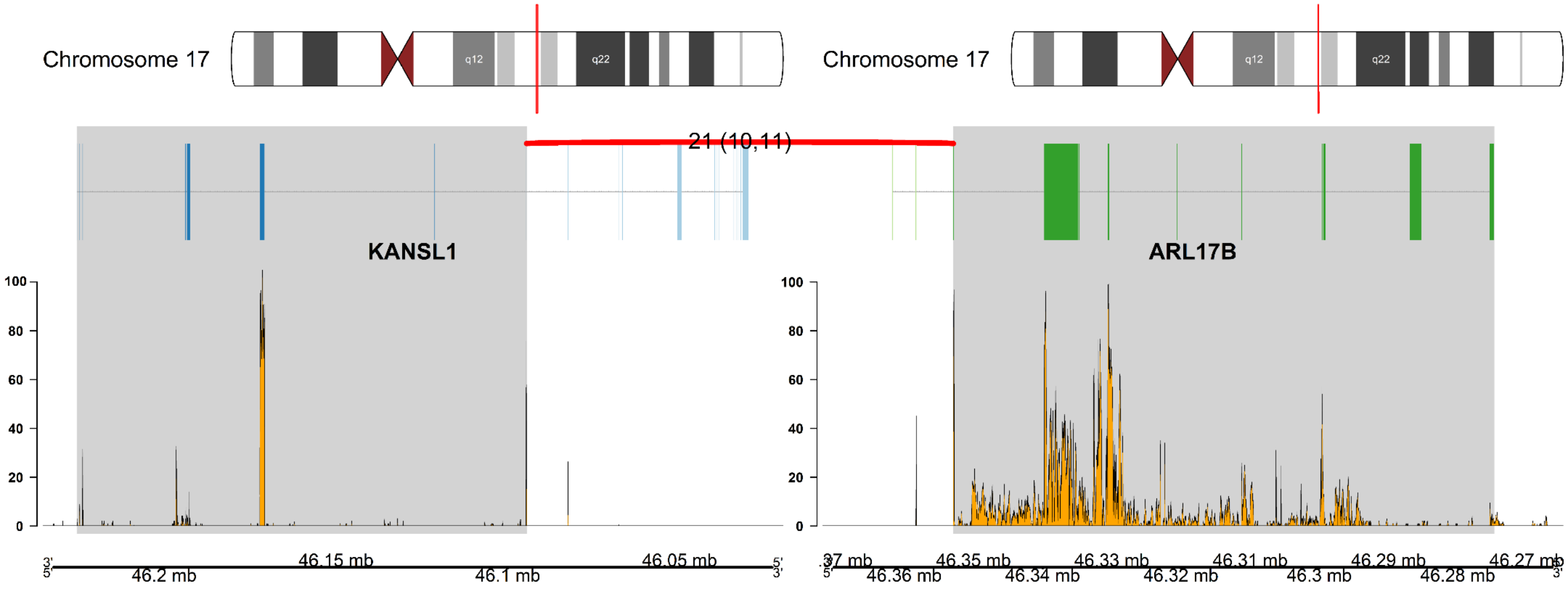

Figure S5

Overview of fusion KANSL1:ARL17B for European American patient 6.

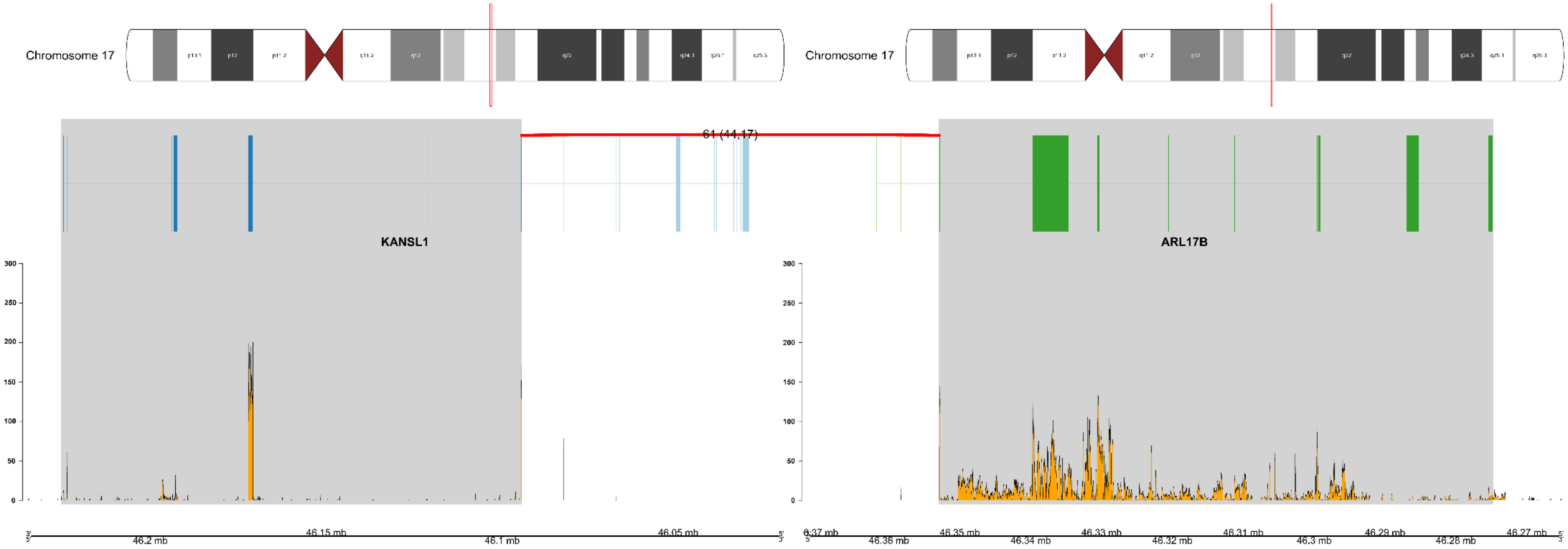

Figure S6

Overview of fusion KANSL1:ARL17B for European American patient 15.

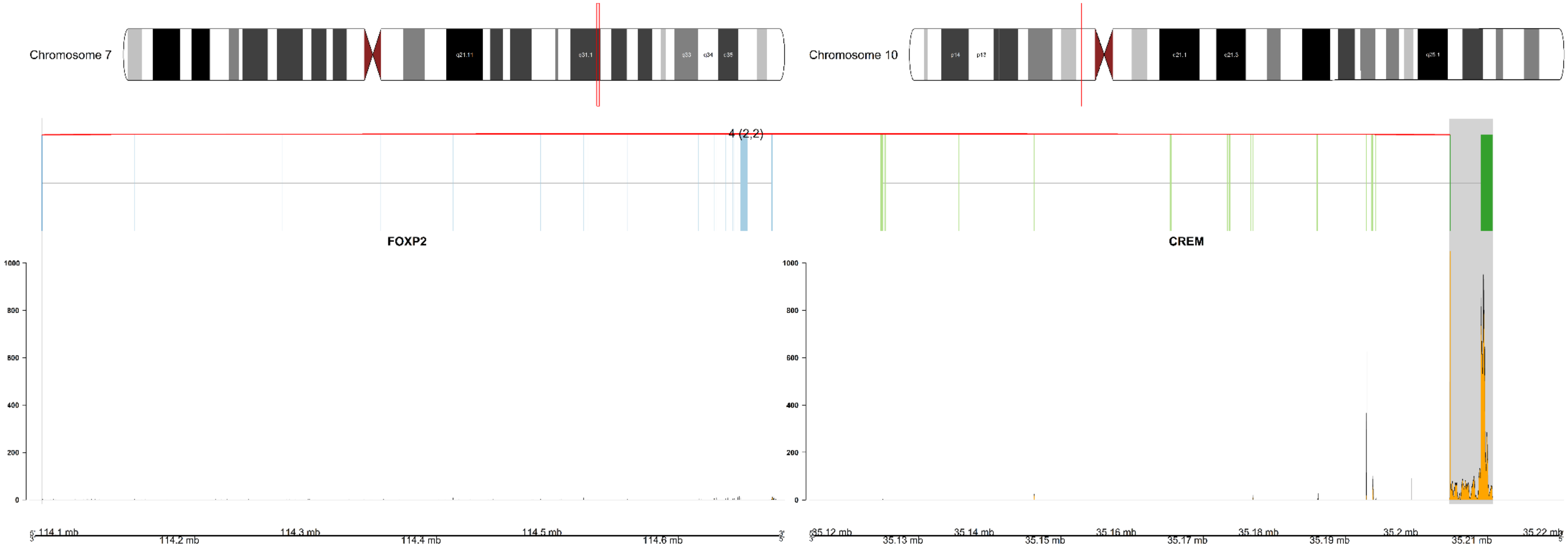

Figure S7

Overview of fusion FOXP2:CREM for European American patient 1.

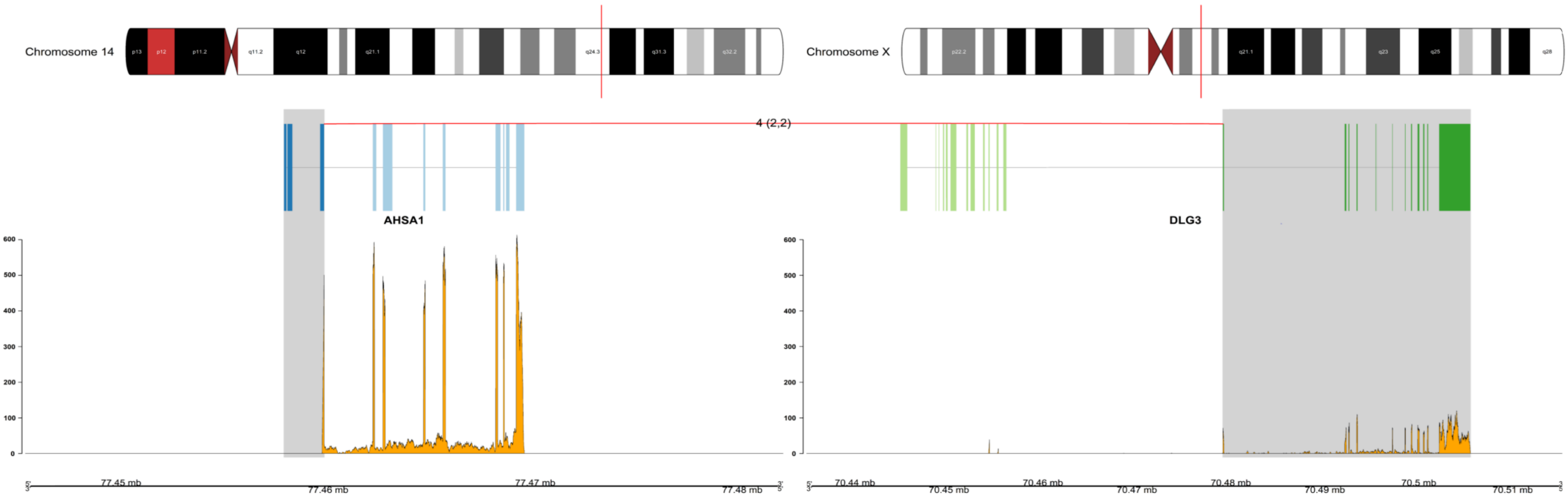

Figure S8

Overview of fusion AHSA1:DLG3 for European American patient 15.
